# Supplementing Maturation Medium With Insulin Growth Factor I and Vitrification-Warming Solutions With Reduced Glutathione Enhances Survival Rates and Development Ability of *in vitro* Matured Vitrified-Warmed Pig Oocytes

**DOI:** 10.3389/fphys.2018.01894

**Published:** 2019-01-14

**Authors:** Barbara Azevedo Pereira, Marcio Gilberto Zangeronimo, Miriam Castillo-Martín, Beatrice Gadani, Bruna Resende Chaves, Joan Enric Rodríguez-Gil, Sergi Bonet, Marc Yeste

**Affiliations:** ^1^Unit of Cell Biology, Biotechnology of Animal and Human Reproduction (TechnoSperm), Department of Biology, Faculty of Sciences, Institute of Food and Agricultural Technology, University of Girona, Girona, Spain; ^2^Laboratory of Animal Physiology and Pharmacology, Department of Veterinary Medicine, Federal University of Lavras, Lavras, Brazil; ^3^Department of Veterinary Medical Sciences, University of Bologna, Bologna, Italy; ^4^Unit of Animal Reproduction, Department of Animal Medicine and Surgery, Faculty of Veterinary Medicine, Autonomous University of Barcelona, Cerdanyola del Vallès, Spain

**Keywords:** growth factors, IGF-I, antioxidants, GSH, apoptosis, cryotolerance, DNA fragmentation, swine

## Abstract

The present study sought to determine whether *in vitro* maturation (IVM) of pig oocytes in a medium supplemented with insulin growth factor-I (IGF-I) and subsequent vitrification with or without reduced glutathione (GSH) affect their quality and developmental competence, and the expression of genes involved in antioxidant, apoptotic and stress responses. In Experiment 1, cumulus-oocyte complexes were matured in the absence or presence of IGF-I (100 ng·mL^−1^) and then vitrified-warmed with or without 2 mM of GSH. Maturation rate was evaluated before vitrification, and oocyte viability, DNA fragmentation and relative transcript abundances of BCL-2-associated X protein (*BAX*), BCL2-like1 (*BCL2L1*), heat shock protein 70 (*HSPA1A*), glutathione peroxidase 1 (*GPX1*) and superoxide dismutase 1 (*SOD1*) genes were assessed in fresh and vitrified-warmed oocytes. In Experiment 2, fresh and vitrified-warmed oocytes were *in vitro* fertilized and their developmental competence determined. Whereas the addition of IGF-I to maturation medium had no effect on oocyte maturation, it caused an increase in the survival rate of vitrified-warmed oocytes. This effect was accompanied by a concomitant augment in the relative transcript abundance of *HSPA1A* and a decrease of *BAX*. Furthermore, the addition of GSH to vitrification-warming media increased survival rates at post-warming. Likewise, the action of GSH was concomitant with an increase in the relative abundance of *GPX1* and a decrease of *BAX* transcript. Blastocyst rates of vitrified-warmed oocytes did not differ from their fresh counterparts when IGF-I and GSH were combined. In conclusion, supplementing maturation medium with 100 ng·mL^−1^ IGF-I and vitrification-warming solutions with 2 mM GSH improves the quality and cryotolerance of IVM pig oocytes, through a mechanism that involves *BAX, GPX1* and *HSPA1A* expression.

## Introduction

Cryopreservation of gametes is considered an efficient tool to maintain genetic resources, contributing to research and development of assisted reproductive technologies (Zhou and Li, 2009; Galeati et al., 2011). However, cryopreservation causes damages to the oocyte structure, chromosomes, and microtubules, including the meiotic spindle (Shi et al., 2006; Wu et al., 2006; Fu et al., 2009). Furthermore, cold shock alters mitochondrial activity, affecting apoptosis pathways (Dai et al., 2015), and also induces premature extrusion of cortical granules, thus reducing sperm penetration and further embryo development (Ghetler et al., 2006).

Pig oocytes are highly sensitive to cooling and freezing due to the large amount of lipids present in the cytoplasm (Galeati et al., 2011), which increase their susceptibly to oxidative stress and lipid peroxidation. Furthermore, vitrified pig oocytes have higher levels of reactive oxygen species (ROS) than their fresh counterparts (Gupta et al., 2010). As a result, developmental competence of pig oocytes is seriously affected by vitrification (Wu et al., 2013; Santos et al., 2017). Therefore, oxidative stress during pig oocyte vitrification and warming results in DNA damage and impairs fertilization and embryonic development (Galeati et al., 2011; Wu et al., 2013; Spricigo et al., 2017).

Glutathione (GSH) is a ubiquitous, major non-enzymatic antioxidant that plays a crucial role as a cellular protector and in the maintenance of intracellular redox status (Hansen and Harris, 2014; Trapphoff et al., 2016). Glutathione may be found in its reduced form (GSH) or oxidized (glutathione disulfide, GSSG), and the GSH:GSSG ratio is used to estimate the redox state of the cell. Such a role is important for the protection of the oocyte against oxidative stress during vitrification (Mari et al., 2009). GSH is synthesized throughout oocyte maturation and its highest concentration is reached at the MII stage. Variations in intracellular GSH content or GSH-related GSH/GSSG redox potential (E_GSH_) can induce post-ovulatory aging, compromise male pronuclear formation, augment apoptosis and impair embryonic development (Li et al., 2012). Somfai et al. (2007) showed that *in vitro* maturation (IVM) and cryopreservation significantly reduce overall GSH content in pig oocytes. Supplementing maturation media with GSH improves mitochondrial function and regulation of redox homeostasis (Trapphoff et al., 2016). Moreover, the addition of GSH to cryopreservation media stabilizes the nucleoprotein structure of frozen–thawed boar spermatozoa (Yeste et al., 2014) and increases blastocyst development of vitrified-warmed mouse oocytes (Moawad et al., 2017).

The composition of *in vitro* maturation media has strong impact on oocyte quality and cryotolerance. In this regard, supplementation of maturation and culture media with insulin-like growth factor I (IGF-I) stimulates oocyte maturation and promotes blastocyst development in several species (Kocyigit and Cevik, 2015; Pan et al., 2015; Arat et al., 2016; Chen et al., 2017). In pigs, although IGF-I has been reported to promote the synthesis of hyaluronic acid and the expansion of cumulus cells (Nemcova et al., 2007), its role in IVM remains unclear and has yield inconsistent results. Nevertheless, several studies reported that the addition of IGF-I during *in vitro* maturation and culture decreases apoptosis in bovine oocytes (Wasielak and Bogacki, 2007; Rodrigues et al., 2016; Ascari et al., 2017) and in porcine blastocysts (Wasielak et al., 2013). Wasielak et al. (2013) also demonstrated that adding IGF-1 at a concentration of 100 ng/ml into maturation medium increases the *BCL2L1:BAX* transcript ratio in pig blastocysts, and the increased expression of anti-apoptotic genes, such as *BCL2*, and a decreased expression of apoptotic-related genes, such as *BAX*, indicates higher blastocyst quality (Chen et al., 2017). Finally, besides reducing apoptosis, IGF-I also enhances the expression of cold-inducible RNA-binding protein (CIRBP), which protects cells from vitrification and warming (Pan et al., 2015).

Against this background, the hypotheses were: (1) adding IVM medium with IGF-I improves the cryotolerance of pig oocytes via anti-apoptotic and heat shock signaling pathways; and (2) supplementation of vitrification–warming media with GSH protects the oocyte from oxidative stress. To test these hypotheses, the present study determined whether supplementing IVM medium with 100 ng/mL IGF-I and adding 2 mM GSH to vitrification–warming media alter the viability, DNA integrity, developmental competence of vitrified-warmed IVM pig oocytes, as well as the relative transcript abundance of genes involved in antioxidant (*GPX1* and *SOD1*), apoptotic (*BAX, BCL2L1*) and stress responses (*HSPA1A*).

## Materials and Methods

### Ethics and Reagents

The present study was carried out after institutional approval by Universidade Federal de Lavras, Brazil (UFLA). All experimental protocols accomplished the Ethical Principles of Animal Experimentation adopted by the Institutional Animal Care and Use Committee Guidelines from this institution (protocol number 043/14).

Unless otherwise specified, all reagents were purchased from Sigma–Aldrich (St Louis, MO, USA).

### *In vitro* Maturation (IVM) of Cumulus–Oocyte Complexes (COCs)

Ovaries were collected from pre-pubertal gilts at a local abattoir (Frigoríficos Costa Brava, S.A.; Girona, Spain) and immediately transported to the laboratory, inside an isolated recipient filled with physiological saline solution supplemented with 1 mg·mL^−1^ kanamycin sulfate and pre-warmed at 38°C. Cumulus–oocyte complexes (COCs) were obtained by aspirating 3–6 mm follicles, using an 8-gauge needle attached to a 10 mL disposable syringe. In each replicated, approximately 50 ovaries, from 25 different gilts, were aspired. After follicle aspiration, the conical tubes containing the aspirated fluid were rested in a water bath at 38.5°C for 10 min. After this time, the supernatant was removed to produce the follicular fluid necessary for supplementation of the *in vitro* maturation media. Additionally, the pellet containing the COCs were transferred to a Petri dish (35 mm, Nunc, Denmark) prefilled with 3 mL Dulbecco's phosphate-buffered saline (DPBS) containing 1 mg·mL^−1^ polyvinyl alcohol (PVA), and then selected under a stereomicroscope. Only COCs with complete and dense cumulus oophorus and homogenous cytoplasm were used. After three washes in the same medium, groups consisting of 50 COCs were transferred into a Nunc 4-well multidish containing 500 μL of modified North Caroline State University 37 (NCSU37) medium (Petters and Wells, 1993), supplemented with 0.57 mM cysteine, 5 mg·mL^−1^ insulin, 50 μM β-mercaptoethanol and 10% (v/v) porcine follicular fluid (PFF). COCs were cultured at 38.5°C in a humidified environment with 5% CO_2_ content in the air. The medium used for the first 22 h of *in vitro* maturation was supplemented with 1 mM dibutyrylcAMP (dbcAMP), 10 IU·mL^−1^ equine chorionic gonadotropin (eCG; Foligon; Intervet International, Boxmeer, Netherlands) and 10 IU·mL^−1^ human chorionic gonadotropin (hCG; Chorulon; Intervet International, Boxmeer, Netherlands). After 20–22 h of culture, COCs were transferred into fresh maturation medium and cultured for a 20–22 h period without any supplementation (Funahashi et al., 1997). Besides the hormonal supplementation traditionally used in the first 22–24 h, half of selected oocytes is maturated in presence of 100 ng.ml-1 of IGF-I, which were added in the maturation media in both periods, in other words, the IGF-I supplementation was made in the entire process.

#### Evaluation of *in vitro* Maturation

**Figure 1:**
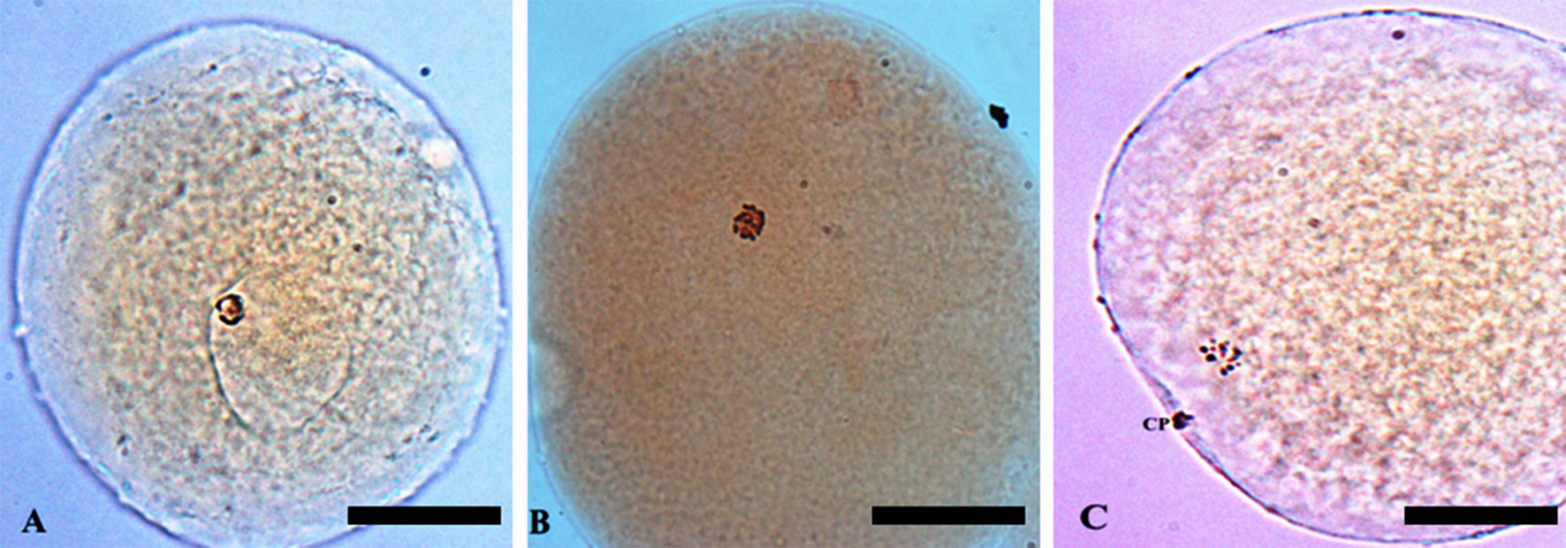
Classification of porcine oocytes using the orcein staining method: **(A)** germinal vesicle (GV); **(B)** metaphase I (MI); and **(C)** metaphase II (MII) stage. Magnification of 100x. Bar: 65 μm. *CP, polar corpuscle*.

*In vitro* maturation of oocytes was evaluated by orcein staining (Hunter and Polge, 1966). Briefly, oocytes were mounted onto glass slides (less than five oocytes per slide) under coverslip (supported with paraffin-vaseline corners) and fixed in ethanol: acetic acid (3:1; v: v) for 24 h. Then, oocytes were stained with 1% orcein (w: v) in 45% acetic acid (v: v) and assessed using a phase-contrast microscope at 100x magnification. Oocytes were classified according to the stage of nuclear maturation as germinal vesicle (GV), metaphase I (MI) and metaphase II/telophase I (MII/TI) (Figure 1).

### Oocyte Vitrification and Warming

*In vitro* matured oocytes were vitrified using the Cryotop carrier and the solution described by Kuwayama et al. (2005). All manipulations were performed on a hot plate at 38.5°C under a laminar flow hood in a room at 25°C. Briefly, immediately after *in vitro* maturation, presumptive maturated denuded oocytes were transferred into equilibration solution (ES) consisting of 7.5% ethylene glycol (EG) and 7.5% dimethylsulfoxide (DMSO) in a holding medium, composed of TCM-199 HEPES and supplemented with 20% fetal calf serum (FCS; GIBCO BRL, Invitrogen, Barcelona, Spain). After 10–15 min at 38.5°C, oocytes were transferred into 20-μL drops of vitrification solution (VS) consisting of holding medium supplemented with 15% EG, 15% DMSO and 0.5 M sucrose. After 30–40 s, oocytes were loaded into a manufactured Cryotop device with minimum volume of vitrification solution and plunged immediately into liquid nitrogen. The entire process, from VS exposure to plunging into liquid nitrogen was completed within 60 s.

Vitrified oocytes were warmed by submerging Cryotop devices directly in thawing solution (holding medium supplemented with 1 M sucrose) at 39°C. After 1 min, oocytes were transferred into dilution solution (0.5 M sucrose dissolved in holding medium) for 3 min. Subsequently, oocytes were washed twice for 5 min in TCM-199 HEPES supplemented with 20% FCS and then cultured in plain IVM medium for 2 h.

### *In vitro* Fertilization and Embryo Culture

*In vitro* matured oocytes from all treatment groups were fertilized *in vitro* before and after vitrified-warmed process. Briefly, oocytes were washed twice with pre-equilibrated modified Tyrode's albumin lactate pyruvate (TALP) prior to being transferred in groups of 20 into four-well dishes containing 250 μL of fertilization medium per well. Fertilization medium consisted of TALP medium described by Rath et al. (1999), supplemented with 3 mg·mL^−1^ fatty acid-free bovine serum albumin (BSA) and 1.1 mM sodium pyruvate.

Sperm-rich fractions were collected by the gloved-hand technique from a mature Duroc boar known to be fertile. For *in vitro* fertilization, sperm cells were selected by washing through a two-step (50% and 80%; v:v) Percoll gradient (Noguchi et al., 2013). Briefly, 2 mL of 50% Percoll was layered on top of 2 mL of 80% Percoll in a 15 mL conic centrifuge tube. Thereafter, 0.5 mL of diluted semen (1:1 in BTS) was added with care to avoid mixing solutions. Samples were subsequently centrifuged at 700 × *g* and 24°C for 20 min. Supernatant layers were removed by aspiration; the resulting sperm pellet was re-suspended in 10 mL pre-equilibrated TALP medium and then washed by centrifugation at 500 × *g* and 24°C for 5 min. The resulting pellet was re-suspended in the same medium and sperm concentration was determined with a Makler counting chamber (Sefi Medical Instruments; Haifa, Israel). Finally, and after appropriate dilution in TALP medium, 250 μL of sperm suspension was added to each fertilization well containing oocytes to obtain a final concentration of 1 × 10^6^ spermatozoa·mL^−1^.

At 1.5 h post-insemination (h.p.i.), oocytes were washed and settled into culture with fresh TALP medium. After 7.5 h.p.i., extra spermatozoa were removed by repeated pipetting and presumptive zygotes were washed twice in culture medium and then transferred to 25-μL culture droplets (15–20 embryos/drop) under mineral oil (Nidoil; Nidacon, Sweden). The basic culture medium used for embryo development was a modified NCSU-23 medium (Petters and Wells, 1993) supplemented with 0.57 mM cysteine, 5 mg·mL^−1^ insulin, 10 mL·mL^−1^ minimum essential medium (MEM) non-essential amino acid solution, 20 mL·mL^−1^ basal medium Eagle (BME) amino acid solution and 4 mg·mL^−1^ BSA. From Day 0 (the day of IVF) to Day 2, the medium used for embryo culture contained sodium pyruvate (0.17 mM) and sodium lactate (2.73 mM) as energy sources. From Day 2 to Day 7, the basic culture medium contained glucose (5.55 mM; Castillo-Martin et al., 2014a). All incubations were performed at 38.5°C in a humidified environment with 5% CO_2_ in air.

#### Evaluation of Fertilization and Embryo Development

Presumptive zygotes were attached onto glass slides and fixed with the same protocol used to evaluate oocyte maturation. At 18 h.p.i., oocytes were evaluated under a phase-contrast microscope at 100 × magnification and the following parameters were assessed: (1) penetration rate (number of fertilized oocytes/number of inseminated oocytes); (2) monospermy rate (number of oocytes containing only one male-head sperm pronucleus/number of penetrated oocytes); and (3) total efficiency of fertilization (number of monospermic oocytes/number of inseminated oocytes). Degenerated and immature oocytes were not considered. Embryo development was assessed under stereomicroscope based on of cleavage (≥2-cell stage) at 48 h.p.i. and blastocyst rates (number of blastocysts at day 7/number of cleaved embryos).

### Evaluation of DNA Fragmentation and Oocyte Membrane Integrity

To determine plasma membrane integrity and DNA fragmentation of *in vitro* matured fresh and vitrified-warmed oocytes, ethidium homodimer-1 staining (EthD-1) was combined with TUNEL assay (*in-situ* Cell Death Detection System; Roche Diagnostic, Indianapolis, IN, USA) (Fatehi et al., 2005). Briefly, denuded oocytes were rinsed in D-PBS containing 1 mg·mL^−1^ PVA and subsequently incubated with 4 μM EthD-1 (Molecular Probes, Thermo Fisher Scientific; Waltham, MA, USA) in PBS at 37°C in the dark for 5 min. Oocytes were washed in PBS containing 0.3% polyvinylpyrrolidone (PBS–PVP) and then fixed with 4% paraformaldehyde (Electron Microscopy Science, Fort Washington, PA, USA) in PBS at 4°C overnight.

After fixation, oocytes were washed four times in PBS-PVP and permeabilised with 0.1% (v/v) Triton X-100 in PBS for 1 h. Then, oocytes were washed twice in PBS–PVP and incubated in TUNEL reaction cocktail at 37°C for 1 h in a dark and humidified environment. Positive and negative control samples were included in each assessment. Positive control consisted of a previous treatment with DNase I (50 U·mL^−1^) in PBS–PVP at 37°C for 20 min in the dark. Negative control did not contain the terminal transferase. After washing in PBS–PVP, controls and samples were mounted with 4 μL Vectashield Medium (Vector Laboratories, Burlingame, CA, USA) containing 4′, 6-diamidino-2-phenylindole (DAPI, 1.5 mg/ml) on a microscopic slide, and then covered with a coverslip. Stained oocytes were examined under a fluorescence microscope (Zeiss Axio Imager Z1; Carl Zeiss, Oberkochen, Germany), at excitation wavelengths of 365 nm for DAPI, 485 nm for TUNEL (conjugated with fluorescein isothiocyanate, FITC) and 580 nm for EthD-1.

**Figure 2:**
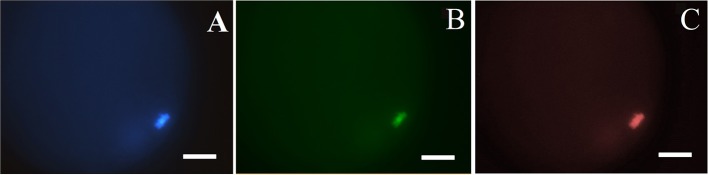
Classification of pig oocytes using simultaneous ethidium homodimer-1 (EthD-1) staining and TUNEL assay: **(A)** DAPI^+^; blue, stains all nuclei; **(B)** TUNEL^+^; green, fragmented DNA; and **(C)** EthD-1^+^; red stain–stains nuclei of non-viable oocytes. Magnification of 400x. Bar: 40 μm.

Total number of oocytes (DAPI^+^; blue), number of non-viable oocytes (EthD-1^+^; red stain) and number of oocytes with fragmented DNA (TUNEL^+^; green) were counted (Figure 2). According to the obtained colorations, oocytes were classified as: (a) viable oocytes with intact DNA (TUNEL^−^/EthD-1^−^); (b) non-viable oocytes with intact DNA (TUNEL^−^/EthD-1^+^) and non-viable oocytes with fragmented DNA (TUNEL^+^/EthD-1^+^).

### RNA Extraction and Quantitative Real-Time PCR Analysis (qPCR)

*In vitro* matured and vitrified-warmed oocytes from all treatments were collected for analysis. Immediately after maturation *in vitro* and vitrification recovery gene expression time denuded oocytes were washed three times in D-PBS containing 1 mg·ml^−1^ PVA at 38.5°C, snap-frozen in liquid nitrogen and stored at −80°C until mRNA extraction and reverse transcription.

Poly(A)-RNA extraction was performed with pools of 20 oocytes per experimental group, following the manufacturer's instructions, using the Dynabeads mRNA Direct Extraction Kit (Dynal Biotech; Oslo, Norway) with minor modifications. In brief, each pool of oocytes was lysed in 50 μL of Lysis buffer for 5 min; the resulting lysate was then hybridized with 10 μL pre-washed beads for 5 min. After hybridization, poly(A)-RNA-bead complexes were washed twice in 50 μL washing buffer A and twice in 50 μL washing buffer B. Next, samples were diluted in 16 μL elution buffer and heated to 70°C for 5 min. Following this, 4 μL qScript cDNA supermix (Quanta Biosciences; Gaithersburg, MD, USA) was added and the Reverse Transcription (RT) reaction was carried by out using oligo-dT primers, random primers, dNTPs and qScript reverse transcriptase. The RT reaction was performed in a thermocycler (Quanta Biosciences; Gaithersburg, MD, USA) at the following conditions: first step of 5 min at 25°C, followed by 1 h at 42°C for RT of mRNA, and 10 min at 70°C to inactivate the RT enzyme. After RT, cDNA was diluted with 25 μL elution solution and stored at −20°C until use.

The relative abundance of mRNA (cDNA) transcripts was determined by real-time quantitative PCR (qPCR) using the 7500 Real Time PCR System (Applied Biosystems; Foster City, CA, USA). The qPCR reaction mix contained 10 μL Fast SYBR Green Master Mix (Applied Biosystems, Foster City, California, USA), 0.25 μL forward primer and 0.25 μL reverse primer (Life Technologies, Madrid, Spain) specific for the genes of interest and 2.5 μL cDNA template. Final volume of 20 μL was reached by adding nuclease-free water. PCR amplification was carried out with one step of denaturation at 95°C for 5 min; 45 cycles of amplification with denaturation step at 94°C for 15 s, annealing step for 30 s at the appropriate annealing temperature for primers; and extension step at 72°C for 40 s. The identity of PCR products was verified with gel electrophoresis (2% agarose gel containing 0.1 μL·mL^−1^ SafeView; Applied Biological Materials, Vancouver, Canada). Three technical replicates per biological replicate and individual gene were evaluated. Furthermore, no-RT control, where the reverse transcription was carried out without RT enzyme, and no template control (NTC), where PCR was conducted without cDNA template, were included for each probe set to ensure that no cross-contamination occurred (i.e., negative controls).

Five separate genes, BCL-2 associated X protein (*BAX*), BCL2-like 1 (*BCL2L1*), heat shock protein 70 (*HSPA1A*), glutathione peroxidase (*GPX1*) and cytosolic copper-zinc-containing superoxide dismutase (*SOD1*), plus an endogenous control gene (glyceraldehide-3-phosphate dehydrogenase, *GAPDH*), were amplified (Table 1). The comparative threshold cycle (C_T_) method was used to quantify relative gene expression levels and quantification was normalized using the endogenous control (*GAPDH*). To determine the threshold cycle for each sample, fluorescence data were acquired after each elongation step. Following the comparative C_T_ method, the ΔC_T_ value was determined by subtracting the *GAPDH*-C_T_ value of each sample from the C_T_ value of each target gene within the sample. Calculation of ΔΔC_T_ involved using the highest sample ΔC_T_ value (i.e., the sample with the lowest target gene expression) as an arbitrary constant to subtract from all other ΔC_T_ sample values. Fold differences in relative transcript abundance were calculated for target genes assuming an amplification efficiency of 100% and using the formula 2${}^{\left(\text{Δ}\Delta C_{\text{T}}\right)}$.

**Table 1:** Primers used for quantitative reverse transcription–polymerase chain reaction.

**NCBI official name (gene symbol)**	**Primer sequence (5^**′**^-3^**′**^)**	**Amplicon size (bp)**	**GenBank Accession no**.
Glyceraldehide-3-phosphate dehydrogenase (*GAPDH*)	F:CTCAACGACCACTTCGTCAA R:TCTGGGATGGAAACTGGAAG	233	NM_00120659.1
BCL2-associated × protein (*BAX*)	F:AACATGGAGCTGCAGAGGAT R:CGATCTCGAAGGAAGTCCAG	204	XM_003127290.2
BCL2-like 1 (*BCL2L1*)	F:GGAGCTGGTGGTTGACTTTC R:CTAGGTGGTCATTCAGGTAAG	528	AF216205
Heat Shock Protein 70 kDA (*HSPA1A*)	F:ATGTCCGCTGCAAGAGAAGT R:GGCGTCAAACACGGTATTCT	216	NM_001123127.1
Superoxide dismutase 1soluble (*SOD1*)	F:GTGCAGGGCACCATCTACTT R:AGTCACATTGCCCAGGTCTC	222	NM_001190422.1
Glutathione peroxidase 1 (*GPX1*)	F:CAAGAATGGGGAGATCCTGA R:GTCA TTGCGACACACTGGAG	217	NM_2142201.1

NCBI, National Center for Biotechnology Information

*F, Forward; R, Reverse; bp, base pairs*.

### Experimental Design

Two experiments were designed to determine whether the addition of IGF-I to conventional IVM medium and that of GSH to vitrification-warming media improved oocyte survival and development capacity after vitrification and warming. Based on previous reports and preliminary experiments conducted in our lab, the treatments tested were IGF-I at 100 ng·mL^−1^ (Wasielak et al., 2013) and GSH at 2 mM (0.62 mg·mL^−1^) (Yeste et al., 2014).

In experiment 1, cumulus–oocyte complexes (COCs) obtained from pre-pubertal gilts ovary were selected and matured *in vitro* in conventional IVM medium (control) or in the same medium supplemented with 100 ng·mL^−1^ IGF-I. For each replicated, around of 50 ovaries were aspirated, and 220 oocytes were selected, which were divided into the two groups previously described and matured *in vitro*. Between 40 and 44 h after the onset of IVM, half of the oocytes in each treatment group (around 55 oocytes) were vitrified and warmed in conventional medium (control vitrified-warmed), or in the same medium added with 2 mM GSH. The other half of oocytes was used to evaluate maturation rates and the quality of non-vitrified IVM oocytes. Before quality evaluations post-vitrification, vitrified-warmed oocytes were incubated for an additional 2-h period in their respective IVM medium and were then collected to assess viability and DNA fragmentation (*n* = 12 replicates/30 oocytes per replicated) and gene expression (*n* = 4 replicates/20 oocytes per replicated).

Additionally, the experiment 2 was conducted for evaluated the influence of IGF-I and GSH on development embryotic capacity of vitrified-warmed oocytes. In this experiment, the process of maturation, vitrification and warming, is the same of experiment 1. However, after 2 h of vitrification recovery, oocytes from each treatment group were *in vitro* fertilized and cultured, and the rates of fertilization, monospermy fecundation, cleavage and blastocyst development were determinate. The number of oocytes evaluated in this experiment was described in Table 2.

**Table 2:** Effects of supplementing IVM medium with IGF-I, and adding vitrified-warmed solutions with GSH on cleavage rates and embryo development.

	**Groups**	**Oocytes**	**D2 Cleaved**	**D7 Blastocyst**	**Blastocyst rate**
		***n***	***n* (%)**	***n* (%)**	**%**
Fresh	Control	163	92 (56.30 ± 1.70) ab	22 (13.49 ± 0.60) a	24.03 ± 0.91 ab
	IGF	167	95 (57.16 ± 1.75) a	29 (17.41 ± 0.56) a	30.56 ± 0.90 a
Vitrified-warmed	Control	95	24 (25.27 ± 1.50) c	5 (5.13 ± 1.72) c	20.00 ± 6.94 b
	IGF	104	34 (31.70 ± 2.38) cd	8 (6.04 ± 1.66) c	18.33 ± 5.09 b
	Control +GSH	98	31 (32.58 ± 1.82) cd	6 (7.50 ± 1.28) c	23.33 ± 4.08 ab
	IGF+GSH	105	36 (34.58 ± 1.82) bd	9 (8.58 ± 1.61) c	24.17 ± 4.72 ab

*Data are shown as mean ± s.e.m. Different letter indicate significant differences between treatments (P < 0.05)*.

*Blastocyst rate = blastocyst/cleaved*.

### Statistical Analyses

All statistical analyses were conducted using a statistical package (IBM SPSS for Windows, Version 23.0; Armonk, NY, USA). All parameters were previously checked for normality and homogeneity of variances (homocedasticity) using Shapiro-Wilk and Levene tests, respectively. When necessary, data were transformed through arcsine square root (arcsin√x).

In experiment 1, the effects of supplementing IVM with IGF-I on *in vitro* maturation were determined with a *t*-test for independent samples. The effects of treatment (i.e., IGF-I, GSH, IGF-I+GSH) and vitrification (i.e., fresh vs. vitrified-warmed) on oocyte viability, DNA fragmentation and relative expression of *BAX, BCL2L1, HSPA1A, GPX1*, and *SOD1* were determined with using a two-way ANOVA followed by Bonferroni *post-hoc* test for multiple comparisons. In experiment 2, the same tests were used and independent variables were penetration, monospermy and cleavage rates, total efficiency of fertilization, and blastocyst formation. When, despite transformed, data did not fit with parametric assumptions, a non-parametric Scheirer-Ray-Hare ANOVA for ranked data was run. The Mann-Whitney test was used for pair-wise comparisons.

Data are expressed as means ± standard error for the mean (SEM). In all cases, *P* ≤ 0.05 was considered as significant.

## Results

### Effects of Supplementing IVM Medium With IGF-I and Vitrification-Warming Solutions With GSH on Oocyte Maturation, Viability and DNA Fragmentation

**Figure 3:**
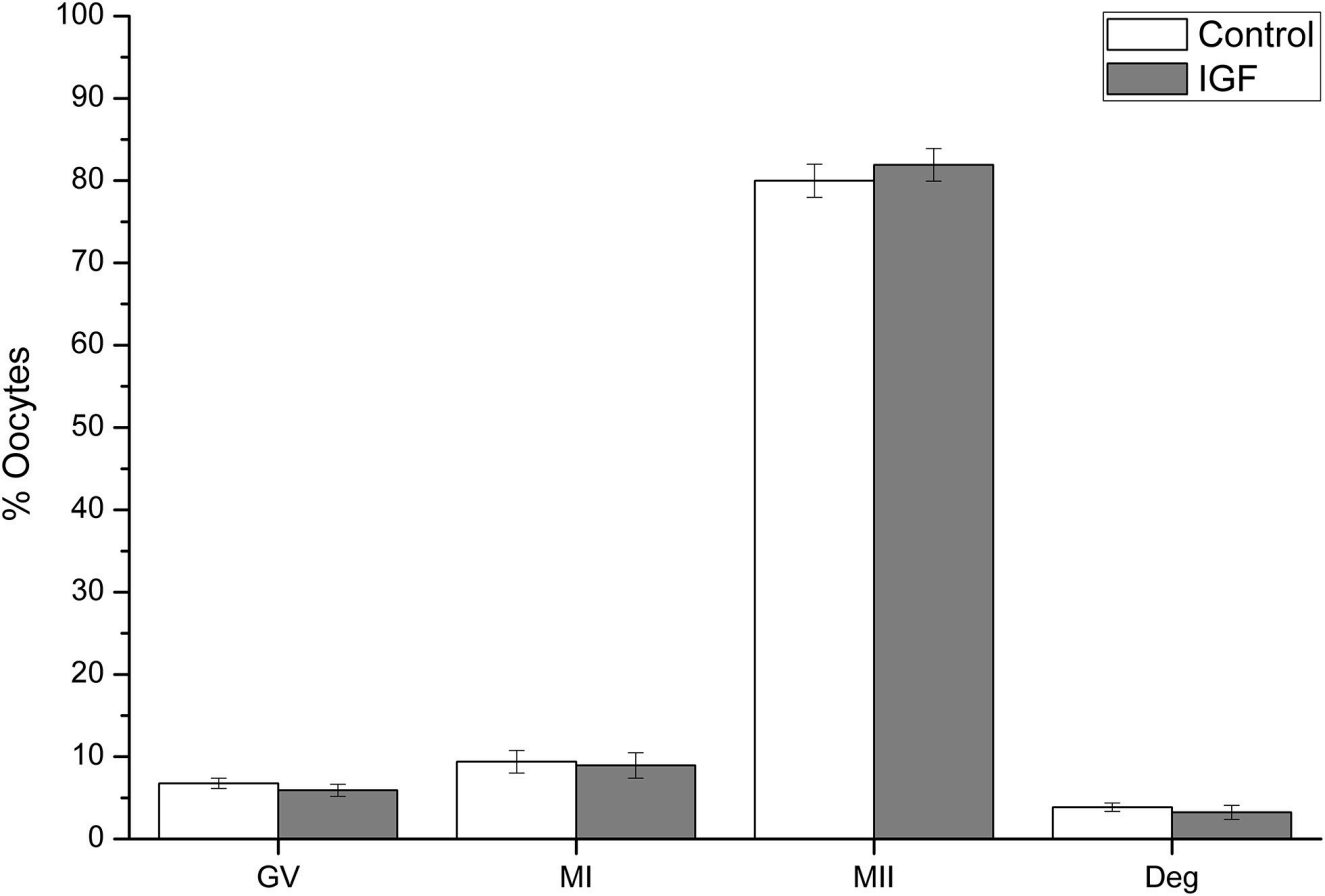
Effects of IGF-I supplementation on *in vitro* maturation of pig oocytes. Data are shown as mean ± s.e.m. GV, Germinal Vesicle; MI, Metaphase I; MII, Metaphase II; Deg, degenerated. No significant differences between control and IGF-I were observed.

A total of 654 oocytes from 12 replicates were evaluated after 44 h of IVM. Supplementation of IVM medium with IGF-I did not affect oocyte maturation, as the proportion of oocytes in all nuclear stages was similar when control and IGF-I groups were compared (Figure 3).

**Figure 4:**
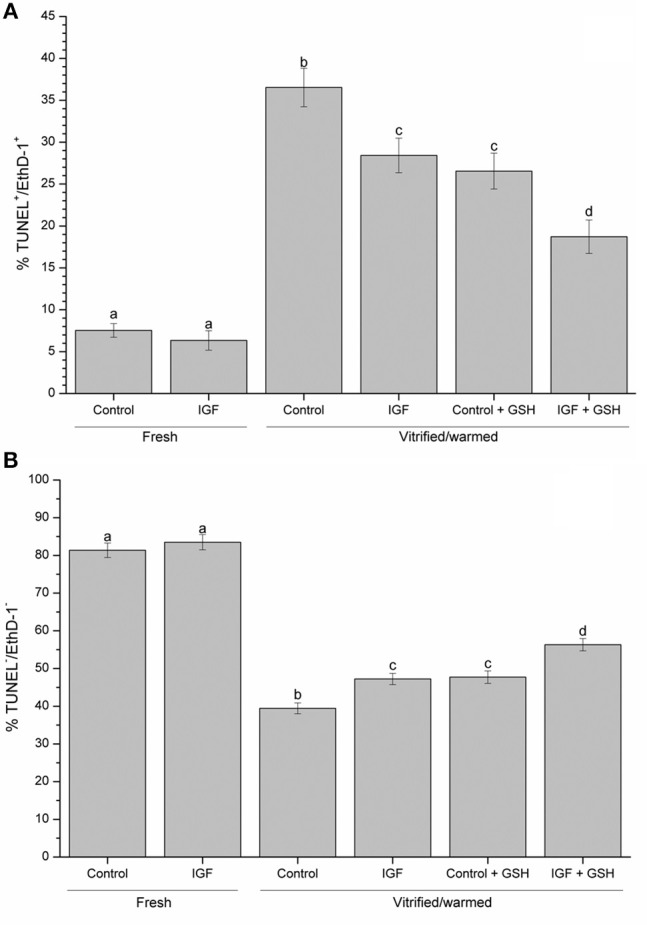
Effects of supplementing IVM medium with IGF-I, and adding vitrified-warmed solutions with GSH on oocyte viability and DNA fragmentation before and after vitrification of porcine oocytes. Data are shown as mean ± s.e.m. Different letters indicate significant differences (*P* < 0.05) **(A)** TUNEL-/EthD-1-: viable oocytes with intact DNA; **(B)** TUNEL+/EthD-1+: non-viable oocytes with fragmented DNA.

With regard to oocyte viability and DNA fragmentation, the percentages of viable, fresh oocytes with intact DNA was significantly higher (*P* < 0.05) than that observed for vitrified-warmed oocytes. Non-vitrified oocytes presented similar viability and DNA fragmentation rates for both treatment groups (i.e. control and IGF-I) (Figure 4).

As far as in vitrified-warmed oocytes, previous *in vitro* maturation in the presence of 100 ng·mL^−1^ IGF-I or supplementation of vitrification-warming solutions with 2 mM GSH (*P* < 0.05) increase the proportion of viable oocytes with intact DNA. Remarkably, the highest proportion of viable vitrified-warmed oocytes with intact DNA was found when they were simultaneously *in vitro* matured in the presence of IGF-I and vitrified-warmed in a medium supplemented with GSH (Figure 4A). The same results were obtained when the proportion of non-viable oocytes to fragmented DNA was evaluated (Figure 4B).

### Effects of Supplementing IVM Medium With IGF-I and Vitrification-Warming Solutions With GSH on the Expression of *BAX, BCL2L1, HSPA1A, GPX1*, and *SOD1*

**Figure 5:**
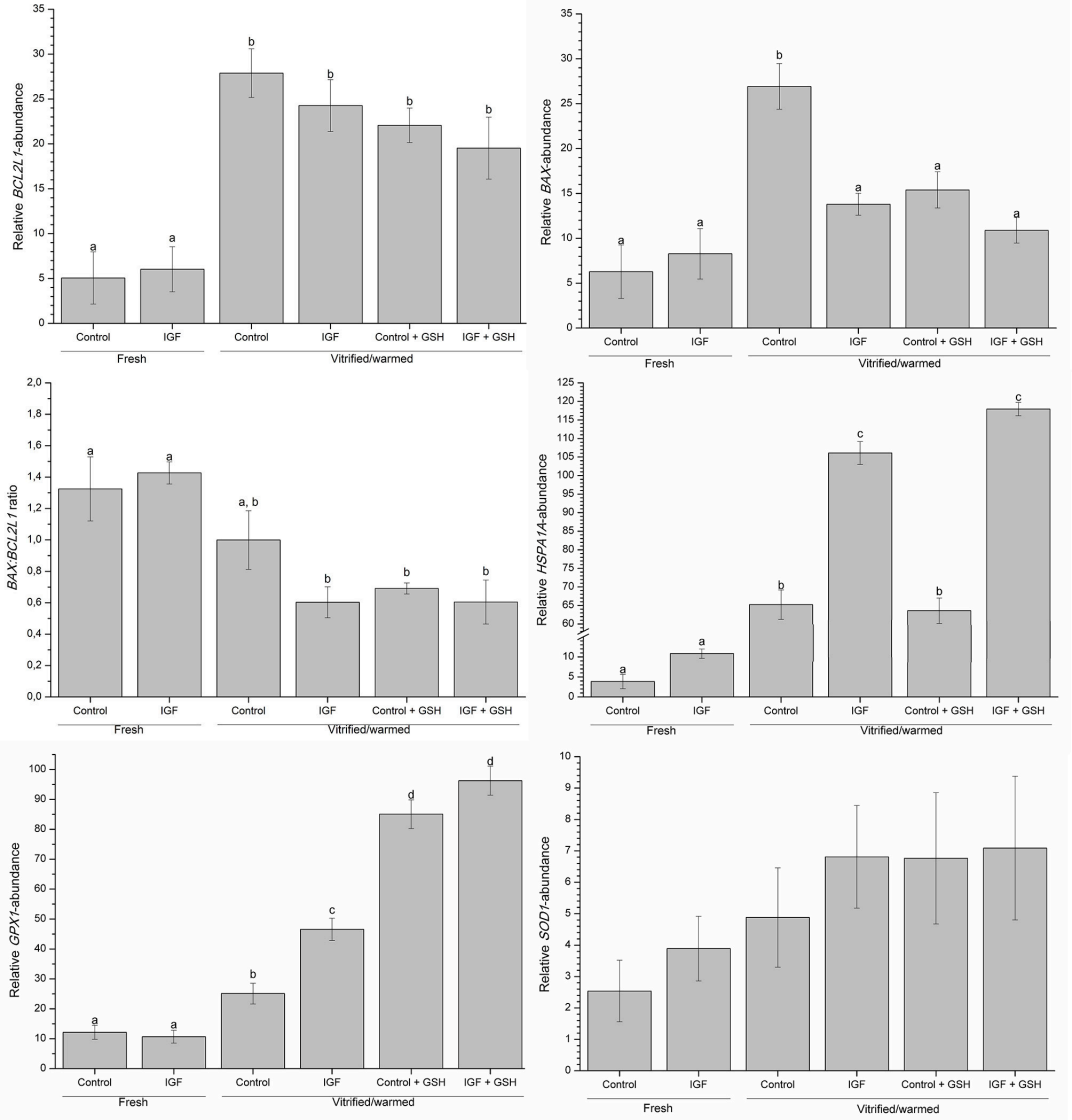
Effects of supplementing IVM medium with IGF-I, and adding vitrified-warmed solutions with GSH on the relative expression of *BAX, BCL2L1, HSPA1A, GPX1* and *SOD1*, and on the *BAX: BCL2L1* ratio before and after vitrification of porcine oocytes. Data are shown as mean ± s.e.m. Different letters indicate significant differences between treatments (*P* < 0.05).

Data regarding relative transcript abundances produced in response to the presence of both IGF-I in IVM medium and GSH in vitrification-warming media are shown in Figure 5. Supplementation of IVM medium with IGF-I had no effect on the expression profiles of *BAX, BCL2L1, HSPA1A, GPX1*, and *SOD1* genes in fresh oocytes. In contrast, supplementing IVM maturation medium with IGF-I and addition of vitrification-warming solutions with GSH (*P* < 0.05) positively affected the expression of *BAX, HSPA1A*, and *GPX1* in vitrified-warmed oocytes but did not alter that of the *SOD1* gene.

A higher expression of *HSPA1A* was observed after vitrification-warming in all treatment groups, but the extent of that increase was significantly (*P* < 0.05) higher in those that were *in vitro* matured in the presence of IGF-I, with or without GSH. On the other hand, while the relative abundance of *GPX1*-transcripts was significantly higher after cryopreservation, supplementing vitrification-warming solutions with GSH led to the highest expression of this gene (*P* < 0.05; see Figure 5). Moreover, relative abundance of *GPX1*-transcripts was also significantly (*P* < 0.05) higher in vitrified-warmed oocytes that had been *in vitro* matured with IGF-I.

Relative transcript abundance of *BCL2L1* gene was significantly (*P* < 0.05) higher in vitrified-warmed oocytes than the fresh oocytes, regardless of supplementing IVM and vitrification-warming media with IGF-I and GSH, respectively. Conversely, although relative *BAX*-transcript abundance was significantly (*P* < 0.05) higher in control vitrified-warmed oocytes, previous *in vitro* maturation with IGF-I and addition of vitrification-warming solutions with GSH did counteract that increase (Figure 5). Therefore, the level of transcripts for the BAX gene was similar between fresh and vitrified-maturated treated oocytes. Furthermore, the *BAX:BCL2L1* ratio was significantly (*P* < 0.05) higher in fresh oocytes than in those vitrified after maturation with IGF-I or vitrified with GSH (Figure 5).

### Effects of Supplementing IVM Medium With IGF-I and Vitrification-Warming Solutions With GSH on Monospermy and Penetration Rates, and on Embryo Development

**Figure 6:**
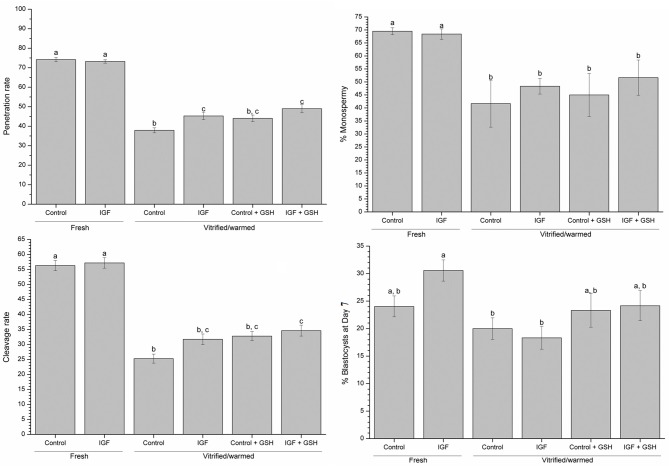
Effects of supplementing IVM medium with IGF-I, and adding vitrified-warmed solutions with GSH on penetration, monospermy, cleavage and blastocysts rates before and after vitrification of porcine oocytes. Data are shown as mean ± s.e.m. Different letters indicate significant differences between treatments (*P* < 0.05). % blastocysts at day 7 = number of blastocyst at day 7: number cleavage oocytes.

Addition of IVM with IGF-I did not influence monospermy, penetration, cleavage and blastocyst rates in embryos derived from fresh oocytes (Figure 6). Monospermy, penetration and cleavage rates, as well as the blastocyst development, were significantly (*P* < 0.05) lower in embryos derived from vitrified-warmed oocytes than in those derived from their fresh counterparts (Table 2). However, penetration rates of vitrified-warmed control oocytes were significantly (*P* < 0.05) lower than those previously matured in the presence of IGF-I (Figure 6). Furthermore, when assessing IVF rates for vitrified-warmed oocytes, cleavage rates were significantly (*P* < 0.05) higher in oocytes previously matured with IGF-I and vitrified-warmed with GSH than in control oocytes (Figure 6). Conversely, neither the addition of IVM medium with IGF-I nor supplementing vitrification-warming solutions with GSH influenced monospermy rates.

Regarding embryo development, the total number of blastocysts formed was negatively affected by the vitrification-warming technique, regardless of the composition of IVM and vitrification-warming media (Table 2). Nevertheless, when blastocyst rates were evaluated, that is, the ratio of blastocyst formed to cleavage oocytes (% blastocyst at day 7), significant (*P* < 0.05) differences between fresh and vitrified-warmed oocytes groups were found. Indeed, fresh oocytes matured with IGF-I gave significantly (*P* < 0.05) higher blastocyst rates than oocytes vitrified-warmed without GSH (Figure 6). In spite of the addition of IGF-I to the maturation medium and GSH in the vitrification and warming media did not improve the rate of cleavage and percentage of blastocyst formed, in making the relation between these two parameters, vitrified-warmed oocytes in the presence of GSH presented blastocyst rate similar to the group of fresh oocytes matured with IGF-I (Table 2).

## Discussion

Our results suggest that supplementing maturation medium with IGF-I and vitrification-warming solutions with GSH improve the quality and cryotolerance of IVM pig oocytes. Moreover, addition of IGF-I to IVM medium and/or of GSH to vitrification-warming solutions also has some beneficial effect on survival rates and developmental competence, and affects the relative transcript abundance of genes related to apoptosis and heat stress. These results are important, as oocyte cryopreservation is known to decrease cell viability and DNA integrity, as well as the cleavage and blastocyst rates of embryos derived from these oocytes. Therefore, a modified composition of IVM and vitrification-warming media with IGF-I and GSH, respectively, appears to better preserve *in vitro* matured pig oocytes. This is especially important as, because of the composition of their cytoplasm and plasma membrane, oocyte cryopreservation in pigs is considered to be more difficult than in other mammalian species (Galeati et al., 2011).

With regard to the addition of IGF-I to IVM medium, it is worth noting that previous studies have reported its positive effect on follicular cell proliferation, oocyte maturation and steroidogenesis (Nemcova et al., 2007; Mani et al., 2010; Xie et al., 2016; Sato et al., 2018). However, our results show that nuclear maturation rates of pig oocytes matured in the presence of 100 ng·mL^−1^ IGF-I do not differ from the control group. Other studies can help us explain these results. Oberlender et al. (2013b) divided recovered pig oocytes into two groups: (1) oocytes from small follicles (2–4 mm) and oocytes from large follicles (5–8 mm), and submitted them to *in vitro* maturation in the presence of IGF-I. These authors observed that the addition of IGF-I at concentrations around 100 ng·mL^−1^ to IVM medium increased the maturation rates of small follicles. In contrast, IGF-I had no effect on IVM when used on large follicles. These data indicate that during follicular growth, changes crucial for oocyte maturation occur in cumulus cells and in the factors present in the follicular fluid. As high quality oocytes from 3 to 6 mm follicles were used in this experiment, and the average maturation rate obtained was higher than 80%, regardless of IVM media composition, which is considered as good (Zhang et al., 2012), the effects of IGF-I on oocyte maturation could have been not apparent enough. In addition, the fact that IGF-I concentration in pig follicles with 4 mm or larger is about 171 ng·mL^−1^ (Oberlender et al., 2013a) could explain why there were no significant effects of IGF-I on IVM rate.

Besides the direct influence on oocyte maturation, the anti-apoptotic effect of IGF-I leads to hypothesize that supplementing maturation media with this growth factor may increase oocyte cryotolerance. As expected, we observed that vitrification-warming significantly affected the viability and DNA integrity of pig oocytes. However, when these cells had been previously matured in a medium supplemented with IGF-I, the percentage of viable oocytes with intact DNA after vitrification-warming was higher than that observed for oocytes matured and vitrified in standard media. These results match with previous studies conducted with pig blastocysts in which supplementing culture medium with IGF-I was found to reduce DNA fragmentation and apoptosis indexes (Dhali et al., 2011; Ascari et al., 2017) by downregulating the expression of *BAX* and upregulating that of *BCL-2* (Wasielak et al., 2013; Pan et al., 2015). To explain these results, one should bear in mind that *BAX* is a pro-apoptotic gene involved in cell death and oocyte degeneration. Following death stimuli (temperature, toxicants or oxidative stress), the *BAX*-linked pathway is activated and the apoptosis cascade is initiated (Finucane et al., 1999). In this study, the addition of IGF-I to oocyte maturation medium significantly reduced the relative abundance of *BAX* transcripts after vitrification-warming, the levels being similar to those found in fresh oocytes. The apoptotic process is related with the balance between pro- and anti-apoptotic genes. While *BAX* family indicates the activation of an apoptosis cascade, the members of the *BCL-2* family (anti-apoptotic gene) form heterodimers with apoptotic genes and block their function (Kim et al., 2006). However, no changes in the expression of *BCL2L1* were found, in a similar fashion to that observed for pre-implanted bovine embryos by (Block et al., 2008). In contrast, Kim et al. (2006) evaluated the effects of IGF-I on IVF pig embryos and observed that IGF-I enhanced the expression of *BCL2L1* and decreased that of *BAX*. In addition to the expression of *BAX* and *BCL2L1*, we also calculated the *BAX*: *BCL2L1* ratio, which determines the susceptibility of a cell to apoptosis and determines cell survival or death (Oltvai et al., 1993). In the current study, although no differences were found in *BCL2L1* expression, the *BAX*: *BCL2L1* ratio has the lowest in vitrified-warmed oocytes maturated in presence of IGF-I and/or vitrified with GSH. This suggests that IGF-I is able to modulate the apoptotic response (Laviola et al., 2007) and also supports the protective effect of GSH (Hansen and Harris, 2014).

Vitrified-warmed oocytes matured with IGF-I presented the highest relative transcript abundance of *HSPA1A*. Heat shock proteins (HSPs) play a critical role in the response to environmental stressful stimuli, including the oxidative stress generated during oocyte maturation and embryo culture (Bernardini et al., 2004) and the thermal stress caused by vitrification-warming procedures (Castillo-Martin et al., 2015). HSPs are a set of highly conserved proteins synthesized in response to stress, which act as molecular chaperones to maintain cellular homeostasis. The intracellular response triggered by IGF-I is modulated by IGF-1-like signaling (IIS) pathway (Laviola et al., 2007), which positively regulates the activity of heat shock factor-1 (HSF-1) (Chiang et al., 2012). HSF-1, in turn, upregulates the transcription of genes involved in the heat-shock response, such as *HSPA1A* (Chiang et al., 2012). The present study showed that the relative transcript abundance of *HSPA1A* was higher in vitrified-warmed oocytes, especially in those previously cultured with IGF-I.

Cryotolerance of oocytes does not only depend on their quality, but also on the conditions provided during vitrification and warming processes. These procedures, as well as other stressing factors, have been reported to disturb the oxidation–reduction (redox) status by both decreasing the reduced glutathione (GSH) content and increasing intracellular reactive oxygen species (ROS) levels in mice (Moawad et al., 2017) and pig oocytes (Somfai et al., 2007; Gupta et al., 2010). Intracellular GSH content is positively correlated with oocyte quality (Hara et al., 2014) and this study indicates that pig oocytes that are vitrified-warmed in GSH-supplemented media show better viability and DNA integrity than those vitrified-warmed in standard media. These results agree with previous works in which GSH was used to improve gamete preservation. Trapphoff et al. (2016) observed that supplementing IVM media with GSH increases cryotolerance of mice oocytes by reducing ROS content and protecting the chromosomal structure. Picco et al. (2010) showed that addition of maturation medium with zinc increases intracellular GSH content and DNA integrity of cumulus cells and bovine oocytes, and this has a positive impact on embryo development. Yeste et al. (2014) demonstrated that supplementing cryopreservation medium with 2mM GSH protects the nucleoprotein structure and maintains the viability of frozen-thawed boar sperm. It is important to emphasize that, in this present study, the highest survival rates were found when pig oocytes were previously matured with 100ng·mL^−1^ IGF-I and were vitrified-warmed with 2 mM GSH. Therefore, both substances appear to have a synergistic effect.

The pathway through which GSH protects the oocytes from the damage inflicted by vitrification-warming remains unknown. What is known is that the generation of endogenous GSH in the oocytes is essential for their protection against the oxidative stress and other forms of cellular injury (Somfai et al., 2007; Trapphoff et al., 2016; Moawad et al., 2017). Therefore, and in the light of our results, it is reasonable to suggest that supplementation of vitrification-warming media with GSH partly counteracts the damaging effects of vitrification by preventing lipid peroxidation in the oocyte and removing the excessive ROS in the medium. Additionally, the positive effect of GSH on the viability and DNA integrity of vitrified-warmed oocytes observed in this study could be explained by the increase in the relative transcript abundance of *GPX1* and the decrease in that of *BAX*. Related with this, relative *GPX1*-transcript abundance has been correlated with embryo quality in cattle, with excellent/good blastocysts having higher expression of *GPX1* in comparison with blastocysts of lower quality (Cebrian-Serrano et al., 2013). Castillo-Martin et al. (2014b) found a positive correlation between *GPX1* and survival rates of vitrified-warmed porcine blastocysts at 24 h post-warming. Glutathione peroxidase is involved in the redox balance of cells and is responsible for scavenging hydrophilic peroxide species, such as hydrogen peroxide (H_2_O_2_). Therefore, with an increase in the antioxidant enzyme content, there is a decrease in the intracellular peroxide levels. Moreover, high ROS levels causes injury on mitochondrial function, which can trigger the intrinsic apoptotic pathway in oocytes and granulosa cells (Dai et al., 2015). This helps explain our results as if the increase in relative *GPX1*-transcript abundance is involved in the reduction of intracellular ROS concentration, the activation of the apoptosis cascade would be reduced, which would agree with the observed decrease in the relative *BAX*-transcript abundance.

Our data indicate that neither supplementation of IVM with IGF-I nor addition of GSH to vitrification-warming media improve blastocyst development or cleavage rates of vitrified-warmed oocytes. However, when pig oocytes are matured with IGF-I and vitrified-warmed with GSH, cleavage rates are better than matured oocytes vitrified-warmed in standard medium. Moreover, when oocytes are vitrified-warmed with GSH, the percentage of cleaved embryos that developed into blastocysts is similar to that of vitrified-warmed fresh oocytes, regardless of the composition of maturation medium. Oocyte developmental competence is related to meiotic spindle and chromosomal configuration and vitrified-warmed IVM oocytes show lower blastocyst rates due to meiotic spindle disorganization and the consequent chromosomal dispersion (Fu et al., 2009; Gupta et al., 2010). Trapphoff et al. (2016) showed a protective effect of GSH on spindle organization and chromosome alignment of mice oocytes, and Moawad et al. (2017) found that developmental potential of mice embryos was higher when vitrified with GSH. The spindle could be particularly protected by GSH because higher GSH content prevents oxidation of cysteine sulfhydryl-groups of αß tubulin dimers (Zhang et al., 2012). In spite of this, one should note that the effects of supplementing vitrification-warming media with GSH could differ between species, as (Hara et al., 2014) reported that high content of GSH in matured bovine oocytes does not suppress the high incidence of multiple aster formation or improves embryo development.

In conclusion, supplementation of IVM medium with 100 ng·mL^−1^ IGF-I and addition vitrification-warming solutions with 2 mM GSH improve survival and DNA integrity rate of vitrified-warmed oocytes, and also increases the relative transcript abundances of *HSPA1A* and *GPX1*, and decreases that of *BAX*. When added together to maturation and vitrification-warming media, respectively, IGF-I and GSH increase cleavage rates in comparison of vitrified-warmed oocytes control, and promoted similar values of blastocyst rates (formed blastocyst: cleavage oocytes) between vitrified-warmed oocytes and fresh oocytes. Our results support the relevance of the *in vitro* maturation process for oocyte cryotolerance and demonstrates the suitability of IGF-I and GSH as additives for maturation and vitrification-warming media.

## Author Contributions

BP, MY, MZ, and JR-G designed the experiments. BP, MC-M, BG, and BC carried out the lab work. BP wrote the manuscript with the support from MY. MY and MZ supervised the project. MC-M, SB, and JR-G helped supervise the project.

### Conflict of Interest Statement

The authors declare that the research was conducted in the absence of any commercial or financial relationships that could be construed as a potential conflict of interest.
